# SIMBA: using Kolb’s learning theory in simulation-based learning to improve participants’ confidence

**DOI:** 10.1186/s12909-022-03176-2

**Published:** 2022-02-22

**Authors:** Meri Davitadze, Emma Ooi, Cai Ying Ng, Dengyi Zhou, Lucretia Thomas, Thia Hanania, Parisha Blaggan, Nia Evans, Wentin Chen, Eka Melson, Wiebke Arlt, Punith Kempegowda

**Affiliations:** 1 Georgian-American Family Medicine Clinic “Medical House”, Tbilisi, Georgia; 2 RCSI & UCD Malaysia Campus, Penang, Malaysia; 3 University of Birmingham Medical School, Birmingham, United Kingdom; 4 Royal Glamorgan Hospital, Cwm Taf Morgannwg University Health Board, Ynysmaerdy, Pontypridd, UK; 5 Ninewells Hospital, NHS Tayside, Dundee, UK; 6 College of Medical and Dental Sciences, Wellcome Trust Clinical Research Fellow, Institute of Metabolism and Systems research, University of Birmingham, Edgbaston, Birmingham, B15 2TT UK; 7 Department of Endocrinology, Queen Elizabeth Hospital, University Hospitals Birmingham NHS Foundation Trust, Birmingham, United Kingdom

**Keywords:** Clinician’s confidence, Endocrinology, Kolb’s learning theory, Medical education, Simulation-based learning

## Abstract

**Background:**

Simulation via Instant Messaging- Birmingham Advance (SIMBA) delivers simulation-based learning (SBL) through WhatsApp® and Zoom® based on Kolb’s experiential learning theory. This study describes how Kolb’s theory was implemented in practice during SIMBA adrenal session.

**Methods:**

SIMBA adrenal session was conducted for healthcare professionals and replicated Kolb’s 4-stage cycle: (a) concrete experience—online simulation of real-life clinical scenarios, (b) reflective observation—discussion and Q&A following simulation, (c) abstract conceptualisation—post-session MCQs, and (d) active experimentation—intentions to implement the acquired knowledge in future practice. Participants’ self-reported confidence levels for simulated and non-simulated cases pre- and post-SIMBA were analysed using Wilcoxon Signed-Rank test. Key takeaway and feedback were assessed quantitatively and qualitatively in a thematic analysis.

**Results:**

Thirty-three participants were included in the analysis. A Wilcoxon signed-rank test showed that the SIMBA session elicited a statistically significant change in participants’ self-reported confidence in their approach to Cushing’s syndrome (Z = 3.873, *p* = 0.0001) and adrenocortical carcinoma (Z = 3.970, *p* < 0.0001). 93.9% (*n* = 31/33) and 84.8% (*n* = 28/33) strongly agreed/agreed the topics were applicable to their clinical practice and accommodated their personal learning style, respectively. 81.8% (*n* = 27/33) reported increase in knowledge on patient management, and 75.8% (*n* = 25/33) anticipated implementing learning points in their practice.

**Conclusions:**

SIMBA effectively adopts Kolb’s theory to provide best possible experience to learners, highlighting the advantages of utilising social media platforms for SBL in medical education. The ability to conduct SIMBA sessions at modest cost internationally paves way to engage more healthcare professionals worldwide.

**Supplementary Information:**

The online version contains supplementary material available at 10.1186/s12909-022-03176-2.

## Introduction

Medical education has been evolving over the years, with digital advancement resulting in development of novel teaching methods. Technology assisted learning has become increasingly integral in contemporary medical education, where flexibility and learner-centred teaching methods take precedence, with e-learning being a major modality [1, 2]. E-learning refers to the use of the Internet to enhance knowledge and allows the learning process to transcend geographical boundaries [3]. E-learning can be a strategy to deliver a simulation-based learning (SBL), designed to provide a learner with a concrete experience in a realistic and safe environment, followed by a debriefing to facilitate abstraction and conceptualisation [4, 5]. SBL addresses two important ethical considerations in medical education: (i) replicates real-life scenarios for acquisition of necessary clinical skills and (ii) ensures patient safety [6]. Contrary to the traditional approaches to medical education, such as lecture-based learning (LBL), SBL calls upon the learner’s integrative capacity and trains learners to adapt to the dynamics of variation in the field [7]. Evidence has shown that SBL is superior to LBL in teaching situation awareness [8] and acquisition of critical assessment and management skills [9].

The benefits of SBL and the technologies it could employ are informed and shaped by the educational theories from domains closely related to them. One example is Kolb’s experiential learning theory which is based on the theory of constructivism and states that knowledge results from the process of grasping and transforming experience; hence, each phase of the Kolb’s cycle must be experienced for optimal learning [10]. This learning cycle consists of four phases: (a) concrete experience where the learner participates in an experience such as simulation, (b) reflection on the experience, (c) abstract conceptualisation where the learner considers thoughts and reflections to identify significance of the learning experience and considers what could have been done differently to enhance the outcome, and (d) active experimentation using what was learned to direct future practice [11]. Constructivism supports the idea that learning is a social experience and requires reflection. Recently, social media has been increasingly used in medical education [12], evolving as a potent tool to deliver SBL and provide immediate feedback during SBL.

Simulation via Instant Messaging- Birmingham Advance (SIMBA) is an innovative teaching model designed to construct SBL through WhatsApp® [13]. With its concept, SIMBA is replicating the learning cycle proposed by Kolb’s experiential learning theory. The online simulation is conducted via WhatsApp® and Zoom®. The session starts off by pairing and linking the participant and moderator on WhatsApp®. Both of them are introduced to each other with an ID to ensure anonymity, which we recommend maintaining throughout the session. The moderator starts the session by sending a pre-SIMBA survey. Upon confirmation of completion, moderators initiate the first simulation by sending a set of instructions about how to go through the simulation as an image. This is followed by a volley of text exchange between participant and moderator to solve the case. Participants ask for information pertaining to the case and moderators send them the information as available on the pre-approved transcript. At the end of the online simulation, a discussion combined with Q&A takes place over Zoom® video-conferencing platform. SIMBA session is closed by a post-SIMBA survey shared by the moderator to the participant on WhatsApp® reflecting on the learners plans to bring changes to future practice based on the experience gained throughout the SIMBA session. The results from previous sessions have demonstrated that SIMBA is an effective teaching model to increase participants’ confidence in managing various endocrine and diabetes simulated cases [13].

The pandemic due to coronavirus (COVID-19) disrupted postgraduate teaching and learning significantly [14]. In our region, there was a complete halt of teaching with all available resources and personnel diverted to tackle the pandemic. To enable sustained training during the pandemic, SIMBA conducted its first international, completely virtual session on Adrenal pathologies. Originally, interactive sessions with an expert were delivered as face-to-face meetings. However, SIMBA switched to Zoom® platform to adjust to the pandemic.

In this study, we describe how Kolb’s experiential learning theory is adapted in SIMBA by providing concrete experience and reflective observation throughout the session, and to evaluate the acceptance of the SIMBA and readiness of the participants to apply built on the knowledge gained.

## Methods

The study was conducted in May 2020 by the SIMBA team with the support of the Institute of Metabolism and Systems Research at the University of Birmingham. The session was conducted over the course of 4 h.

### Preparation for the session

**Fig. 1:**
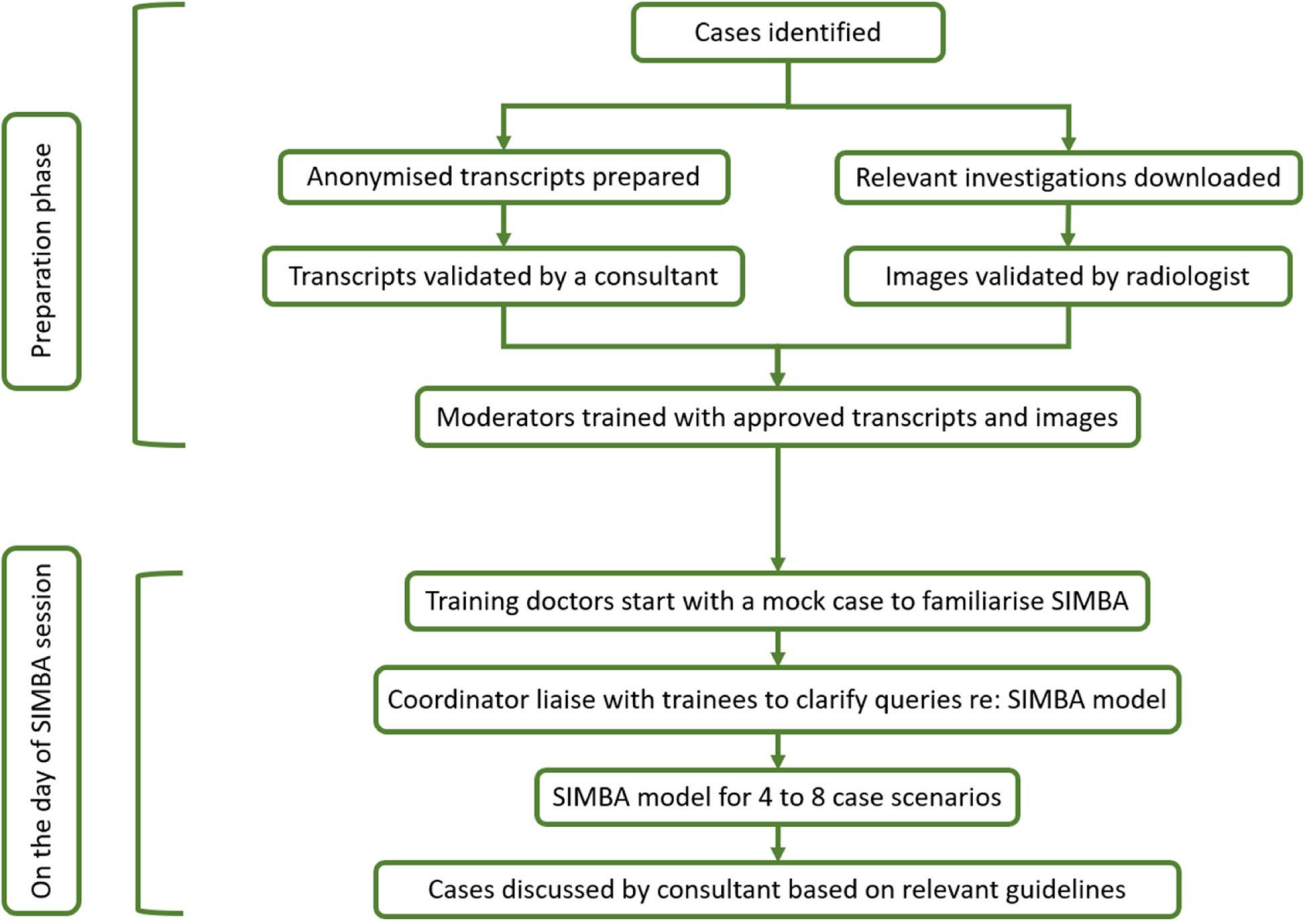
Flowchart of two stages—preparation and on-the-day—of the working model of SIMBA

Standardised transcripts of five anonymised real-life adrenal cases were prepared for the following conditions: adrenal incidentaloma, adrenocortical carcinoma (ACC), Cushing’s syndrome, Conn’s syndrome, and COVID-19 infection in a patient with congenital adrenal hyperplasia (CAH). Each transcript included medical history, clinical examinations, investigation results, imaging studies, management and follow-up plan, and validated by an expert in adrenal pathologies to ensure accuracy of the real-life scenarios. Figure 1 describes in detail the steps building up to SIMBA session and on the day itself.

SIMBA session was facilitated by 29 medical students and 2 junior doctors who volunteered to be moderators for the session. Moderators interacted with the participants throughout the simulation and guided them to solve clinical scenarios. To ensure proficiency, prior to the actual session, all moderators were trained in six mock simulation sessions using the finalised transcripts.

The session was advertised on social media platforms (Facebook® and twitter®) by SIMBA and by endorsing organisations. Interested candidates registered for the session using Google forms. To keep the participants’ identities anonymous, each participant was given a unique identification number (SIMBA ID). A couple of days before the session, participants were emailed their moderator’s contact details and were asked to message their SIMBA ID to their assigned moderator in order to join the session and receive instructions throughout the session (links to pre- and post-SIMBA sessions, and Zoom®) as WhatsApp® was the only platform for communications. Furthermore, the participant must have messaged first in order to protect their personal information.

### On the day of SIMBA adrenal session

#### Pre-SIMBA survey

Each participant was assigned to one moderator, whereas each moderator was guiding two to three participants through the online simulation. After the participants have contacted their moderator, they received a link to pre-SIMBA survey about their socio-demographic information and self-reported confidence in managing various simulated and non-simulated adrenal cases. At this point, the participants were blinded as to which cases would be simulated. Once the submission of pre-SIMBA survey was confirmed by the moderator, simulation was initiated. The detailed description of the SIMBA session is described elsewhere [13]. The simulation provided participants with a concrete experience as per the first phase of the Kolb’s learning cycle.

Following simulation, the participants were invited to a discussion and Q&A of simulated topics by expert endocrinologist with special interest in adrenal pathology via Zoom® to reflect on the concrete experience. The expert discussed the most suitable approach for each case including relevant investigations and management in the context of the most recent evidence-based international guidelines [15–24]. The expert highlighted the most important learning aspects for each case, after which the participants were given an opportunity to ask questions regarding simulated cases or similar clinical scenarios from their past experience.

#### Post-SIMBA survey

After the discussion, participants were invited to complete a post-SIMBA survey, same as the pre-SIMBA survey, but additionally including multiple choice questions (MCQs) related to simulated cases thus enabling abstraction of the knowledge received. To evaluate whether the participants would actively experiment in the future, the survey asked them an open-ended question regarding changes they intend to make in patient care based on the experience gained during the session. Both surveys including the MCQs were developed by the SIMBA team without conducting reliability or validity testing.

### Evaluation of SIMBA

The post-SIMBA evaluation form was designed to obtain feedback from participants regarding its impact. Kirkpatrick’s training evaluation model was adopted, and two outcomes were identified [25]. Level 1 (reaction) was assessed with questions regarding engagement of the session. Level 2 (learning) involved acquiring self-perceived gain in core competencies and confidence levels in approaching various adrenal cases. Participants’ attitude and self-reported confidence (Levels 1 and 2) were captured on 7-point Likert scale due to its simplicity to use and higher validity and reliability compared fewer response categories; additionally, respondents seem to find 7-point scale easier to complete [26]. Open-ended questions were also included in the surveys to explore participants’ intentions to make behavioural changes in patient care following the session. Six domains of medical education based on the core competencies of Accreditation Council for Graduate Medical Education (ACGME) were also assessed using the question “Which of the following competency areas do you feel have been improved by today’s teaching with SIMBA?”, and participants were able to select as many competencies they found relevant (patient care, professionalism, knowledge on patient management, system-based practice, practice-based learning, communication skills).

### Participants’ assessment and feedback

#### SIMBA rating scale

**Fig. 2:**
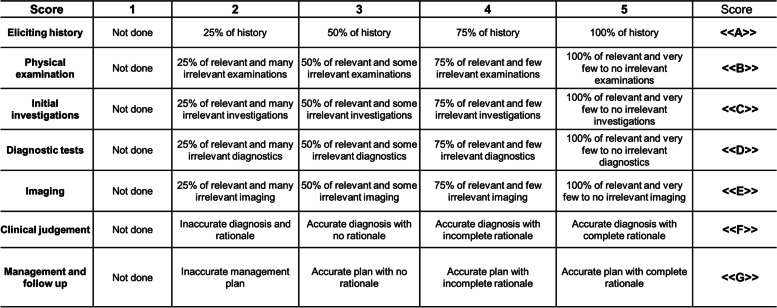
SIMBA rating scale, adapted from the Global Rating Scale by Gerard et al. [27]

Two SIMBA team members independently reviewed the WhatsApp® interaction between the moderator and the participant, and scored the participants’ performance in each simulated case using an adapted version of the global rating scale (Fig. 2) [27]. Seven items (history, examinations, initial investigations, diagnostic tests, imaging, clinical judgement, and management/follow-up) were assessed on a scale of 1 (not done) to 5 (excellent), giving a minimum score of 7 and maximum score of 35. Scores indicate relevance and accuracy of approach to the case in its various components (Fig. 2). The assessment included written feedback for each case based on Pendleton’s model of feedback [28] and the scores were emailed to the participants on the following day of the session.

### Statistical analysis

To capture the differences in confidence levels and explore the impact of the SIMBA session, the adrenal cases included in the questionnaire were divided into two categories: (a) simulated: adrenal incidentaloma > 4 cm, ACC, Conn’s syndrome, Cushing’s syndrome, and COVID-19 infection in a patient with CAH; and (b) non-simulated: bilateral macronodular adrenal hyperplasia (BMNAH), Addison’s disease, secondary adrenal insufficiency, androgen secreting adrenal tumours, adrenal incidentaloma < 4 cm, phaeochromocytoma, and adrenal metastases.

Using agree-disagree Likert scale, we aimed to capture the degree of participants’ confidence. Hence, the data extracted from pre- and post-SIMBA survey were categorised into 3 groups: (i) confident: for those who responded with “strongly agree” and “agree”; (ii) unsure: for those who responded with “agree somewhat”, “undecided”, and “disagree somewhat”; (iii) not confident: for those who responded with “strongly disagree” and “disagree”. Only those participants who completed both pre- and post-SIMBA evaluation forms were included. A Wilcoxon signed-rank test was used to statistically investigate differences in confidence levels between simulated and non-simulated cases of matched pairs pre- and post-SIMBA; performed using Stata (Stata/SE 16.0). Statistical significance was accepted at 95% confidence level (significance set at *p* < 0.05). The change in confidence levels of managing cases pre- and post-SIMBA are reported as percentages and presented in bar charts.

Additionally, in the post-SIMBA evaluation form, participants were asked close- and open-ended questions for feedback and key takeaway from the SIMBA Adrenal session. Findings from close-ended responses are reported in frequencies and percentages. Material collected from open-ended questions were analysed using a single coder using Braun and Clarke method of thematic analysis. Responses from the free text sections were first read and familiarised, systematically identifying main points of the text and attaching labels/codes to capture the main ideas. Relevant and recurrent codes were then collated into themes inductively (data-driven themes) and reviewed. All themes identified are presented in the table. Examples were chosen to encompass the various perspectives, leaving out repetition.

## Results

Out of 40 participants, 33 (82.5%) completed both pre- and post-SIMBA surveys and were included in the analyses. This includes 18 (54.5%) participants from the UK (West Midlands (*n* = 13), London (*n* = 1), North West (*n* = 2), and one participant did not complete specific location data), and 15 (45.5%) internationally (Bosnia and Herzegovina (*n* = 1), Cote d’Ivoire (*n* = 2), Georgia (*n* = 4), India (*n* = 1), Ireland (*n* = 2), Spain (*n* = 2), Syria (*n* = 1), Turkey (*n* = 1), and Ukraine (*n* = 1)). These participants comprise of consultants (*n* = 6), specialist (*n* = 1), senior residents/fellows (*n* = 6), specialty trainee registrars (*n* = 15), resident physicians (*n* = 3), medical doctor (*n* = 1), and currently out of training programme (*n* = 1).

### Participants’ confidence levels

**Fig. 3:**
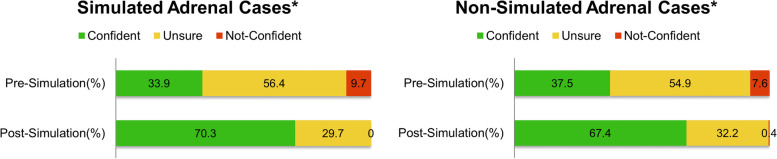
Illustration of changes in participants’ confidence levels for managing simulated vs. non-simulated adrenal cases. *p < 0.0001

A Wilcoxon signed-rank test demonstrated SIMBA session elicited a statistically significant improvement in participants’ self-reported confidence in their approach to simulated cases, as well as in non-simulated conditions (Table 1, Fig. 3). No statistically significant improvement was observed when participants were assessed for their confidence in approach to Addison’s disease (Z = 1.414, *p* = 0.2891).

**Table 1:** Changes in participants’ confidence levels post-SIMBA session for approaching simulated and non-simulated cases with *p* values

Case	Confident	Unsure	Not confident	Significance
**Simulated cases**				
Adrenal incidentaloma > 4 cm	+ 30.3%	− 21.3%	−9.1%	Z = 3.152, *P* = 0.0020
ACC	+ 45.5%	− 33.4%	−12.1%	Z = 3.970, *P* < 0.0001
Conn’s syndrome	+ 36.3%	−27.3%	−9.0%	Z = 3.873, *P* < 0.0001
Cushing’s syndrome	+ 39.3%	−36.3%	−3.0%	Z = 3.742, P = 0.0001
COVID-19 infection in a patient with CAH	+ 30.3%	−15.1%	−15.2%	Z = 3.036, *P* = 0.0029
**Non-simulated cases**				
BMNAH	+ 45.5%	−39.4%	−6.1%	Z = 4.123, *P* < 0.0001
Addison’s disease	+ 12.1%	−12.1%	0.0%	Z = 1.414, *P* = 0.2891
Secondary adrenal insufficiency	+ 24.2%	−18.1%	−3.0%	Z = 2.326, *P* = 0.0332
Androgen secreting adrenal tumours	+ 39.3%	−24.2%	−15.2%	Z = 3.847, *P* < 0.0001
Adrenal incidentaloma < 4 cm	+ 30.3%	−21.3%	−9.1%	Z = 3.152, *P* = 0.0020
Phaeochromocytoma	+ 33.3%	−30.3%	− 3.1%	Z = 3.464, *P* = 0.0005
Adrenal metastases	+ 33.3%	−15.1%	−18.2%	Z = 3.989, *P* < 0.0001

*ACC* Adrenocortical carcinoma, *COVID-19* Novel coronavirus disease, *CAH* Congenital adrenal hyperplasia, *BMNAH* Bilateral macronodular adrenal hyperplasia

### Participants’ feedback/satisfaction and key takeaway

**Fig. 4:**
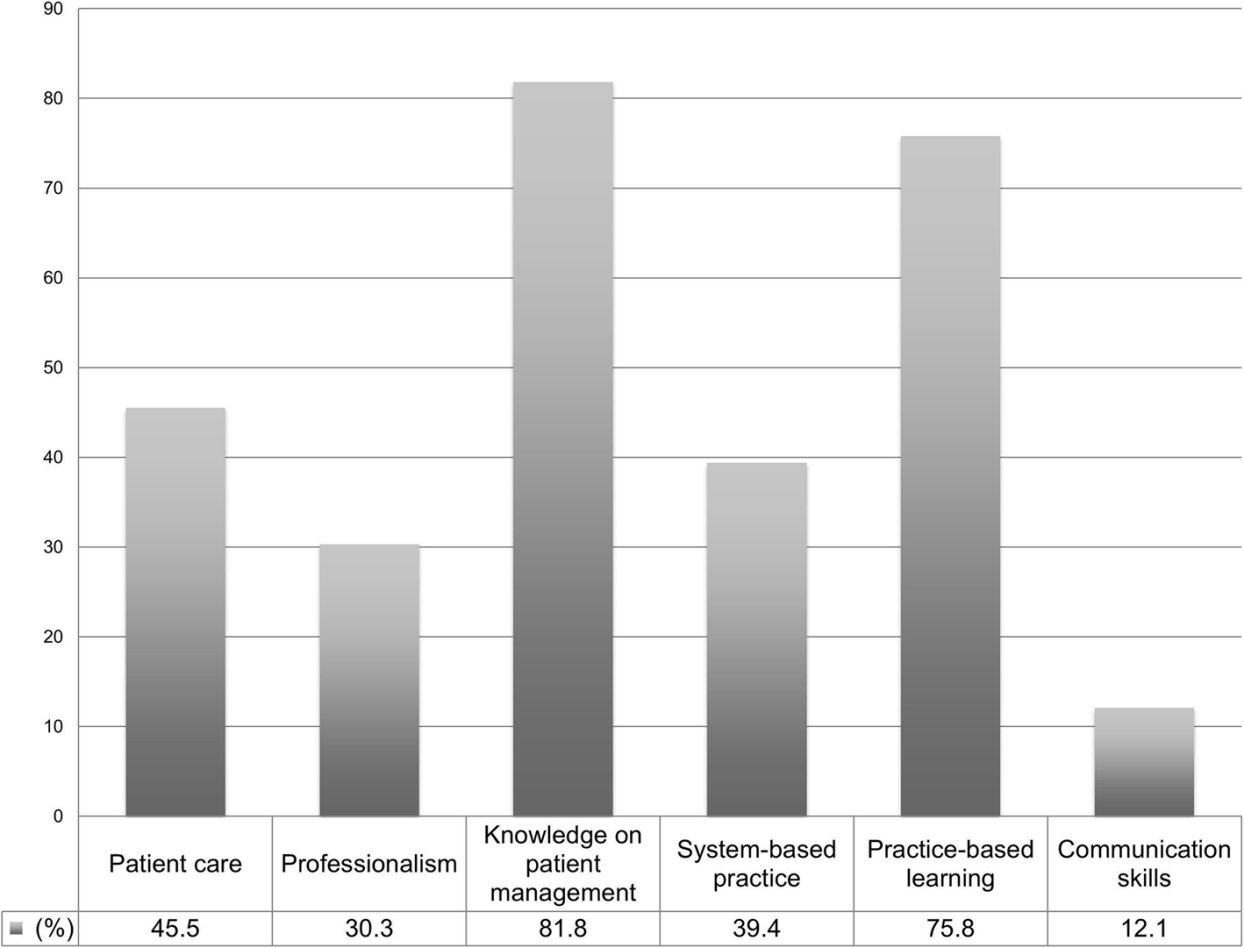
Illustration of changes in Six domains of medical education based on the core competencies proposed by Accreditation Council for Graduate Medical Education (ACGME)

91.1% (*n* = 30/33) strongly agreed/agreed the session was engaging. 84.9% (*n* = 28/33) reported the session accommodated their personal learning style. 97.0% (*n* = 32/33) agreed the chair provided balanced and evidence-based, where possible, approach to the cases. 93.9% (*n* = 31/33) found the simulated topics applicable to their clinical practice, and the session impactful at a personal level. 97.0% (*n* = 32/33) found the content of the session translatable to patient care. Six domains of medical education based on the core competencies by Accreditation Council for Graduate Medical Education (ACGME) were assessed and seen to improve: knowledge on patient management – 81.8% (*n* = 27/33), practice-based learning – 75.8% (*n* = 25/33), patient care – 45.5% (*n* = 15/33), systems-based practice – 39.4% (*n* = 13/33), professionalism – 30.3% (*n* = 10/33), and communication skills – 12.1% (*n* = 4/33) (Fig. 4).

### Thematic analysis of open-ended questions

**Fig. 5:**
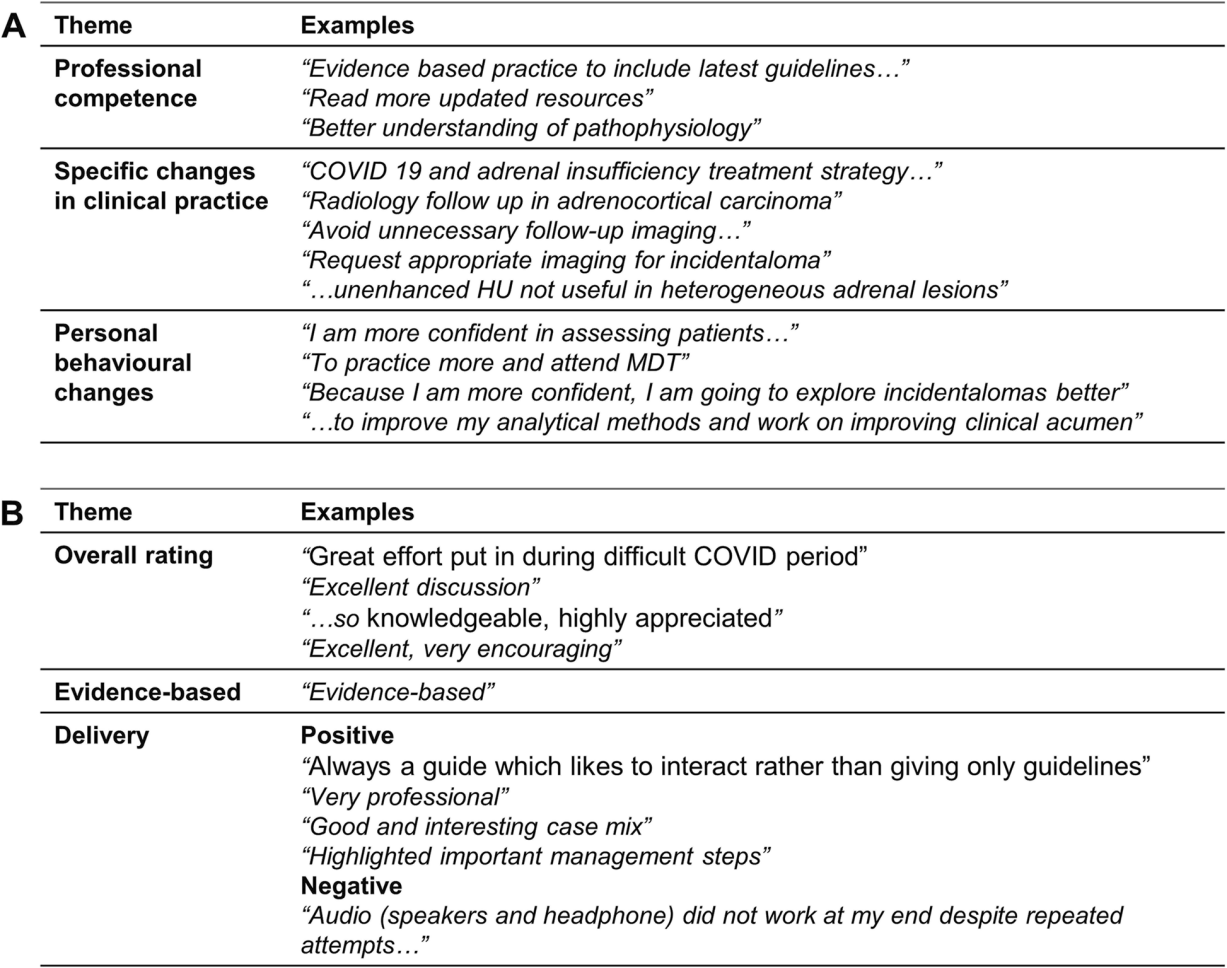
Thematic tabulation of responses to open-ended questions. **A** “as a result of what I have learned today, I intend to make the following changes to my practice that I believe will impact my patients’ care in a positive way”, and (**B**) “additional comments regarding chair’s contribution”

Answers to the open questions were analysed to identify broad themes represented within them. 54.5% (*n* = 18/33) provided a response to the question “as a result of what I have learned today, I intend to make the following changes to my practice that I believe will impact my patients’ care in a positive way”, suggesting a positive influence that can be translated to patient care (Fig. 5A). 36.4% (*n* = 12/33) responded to the open-ended section to provide “additional comments regarding the chair’s contribution”, and 8.3% (*n* = 1/33) was negative, based on technical issues during the session, and the participant was unable to comment on the chair’s contribution. The remaining 33.3% (*n* = 11/33) responses were positive (Fig. 5B).

## Discussion

SIMBA was well received by the participants and proved to be an effective learning model to increase the self-reported confidence level in managing various cases on adrenal pathologies. During a pandemic, moving our SIMBA approach online, combining use of WhatsApp® and Zoom® to deliver the session, played a crucial role in the context of distance learning to provide sustained medical education. This is relevant for the ability of SIMBA to deliver enhanced training across the globe, with minimal resources and low cost.

**Fig. 6:**
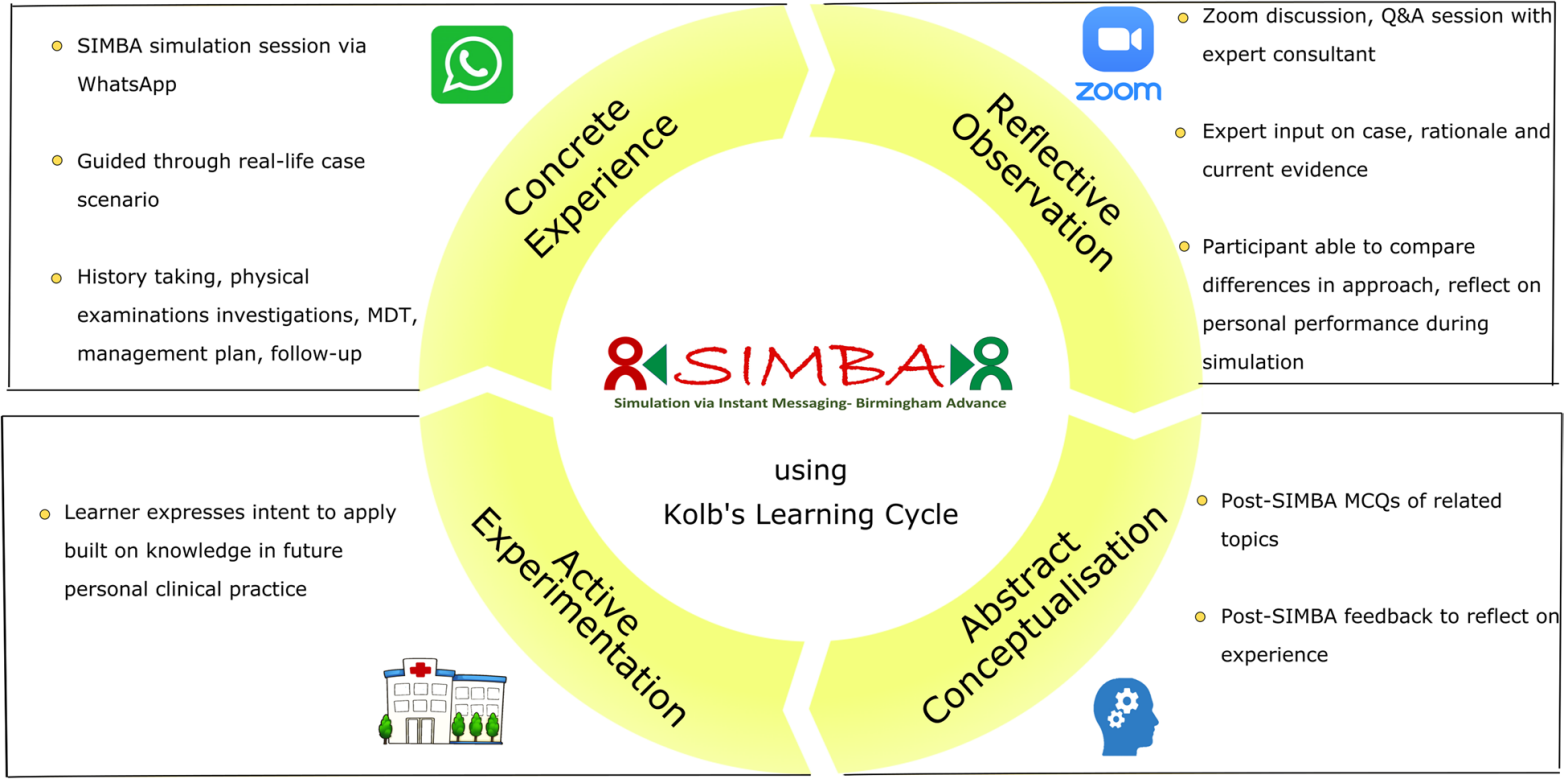
SIMBA replicating Kolb’s 4-stage experiential learning cycle

The results provide insight into participants’ perceived outcomes of the session, demonstrating the Kolb’s 4-stage experiential learning cycle [10], according to which the SIMBA model was constructed (Fig. 6). This process begins with concrete experience—a SIMBA session conducted via WhatsApp®, where the participants were able to work through the motion of realistic case scenarios. Participants’ satisfaction from this session was high, indicating that the model was well-received. The majority of participants found the SIMBA model engaging and accommodating to their personal learning style. This demonstrates that participants from various backgrounds were able to quickly familiarise themselves and adapt with the concept. SIMBA as a model combines e-learning and SBL and evolved with the learning preferences and strengths of the learners of this digital era. This is in line with suggestions to utilise instructions and environments learners are familiar with to improve responsiveness and enhance learning [29, 30]. Further, studies have found that training professionals respond to different learning preferences between generational groups, with younger generations finding greater comfort in technology-based training [30].

The second stage of the cycle—reflective observation—refers to the Zoom® discussion and Q&A session following the simulation. An expert discussed each case with evidence-based rationale. Participants were able to compare what was done differently, reflecting on their personal performance during the simulation. 97.0% of the respondents strongly agreed/agreed that the chair’s contribution was evidence-based, and thematic analysis of feedback indicated positive overall rating. Overall, feedback regarding the delivery of the session demonstrated reflective observation where participants felt “important management steps” were addressed, and that the chair guided the participants through the case explaining current evidence, rather than spelling out the guidelines.

In the third stage of the Kolb’s cycle of abstract conceptualisation, assimilation of the new information was facilitated by the post-SIMBA MCQs on the simulated cases. Throughout the session, participants were able to conceptualise the learnt key points and rationalise/make sense of them, to then add to their existing knowledge. Significant improvements in self-reported confidence levels of simulated cases were observed, and surprisingly, in non-simulated cases as well, likely due to the similarity in topics, requiring a similar approach. Additionally, topics such as phaeochromocytoma were reviewed during discussion which may have influenced participants’ increased confidence levels for non-simulated case scenarios. Feedback on participants’ performance was provided via the SIMBA assessment tool using an adapted Global Rating Scale and Pendleton’s method to allow reflection.

In the fourth stage of the Kolb’s learning cycle—active experimentation—the participant builds on the knowledge in their personal clinical practice. An important finding from this session was the positive impact on participants’ personal and professional learning, potentially translating to personal knowledge and patient care. This matches patient management and practice-based learning components of the ACGME’s core competencies. Practice-based learning refers to achieving recertification following initial certification through continued education in the midst of daily clinical practice, taking into account the constantly evolving nature of the latter [31]. This supports the aims of the SIMBA model, which employs the theory of connectivism, to disseminate latest evidence on the management of patients in the topic, while guiding participants through a scenario to allow subsequent self-appraisal according to scientific evidence, in a safe environment. Additionally, participants responded with intentions to improve aspects of their professional competence and to implement specific changes in clinical practice and personal behaviour. These can be anticipated to have long-term positive consequences in clinically-relevant settings, with a perceived ability to improve patient care.

### Limitations and future research

While providing beneficial insight and supporting evidence, the study has certain limitations. While we were able to assess the effectiveness of SIMBA model using Kirkpatrick’s levels 1 (reaction) and 2 (learning) in our pre- and post-SIMBA evaluation forms, levels 3 (behaviour) and level 4 (results/outcomes) translating to behavioural changes and longitudinal measurement of our model’s impact remains a challenge. Our evaluation is currently limited to self-reported perceptions of improvement and willingness to apply the acquired knowledge and skills to the clinical practice. However, intentions may fail to be translated to actions due to intention-to-action gap [32]. Another limitation of the study is that the measures used were researcher-developed, without evidence of validity and reliability, and dataset for thematic analysis was small. Further longitudinal evaluation is needed to determine whether actual clinical competence is subsequently improved. Another potential line of research would be to investigate the cognitive outcomes such as knowledge and retention of information, in addition to the current non-cognitive outcomes evaluated (self-reported confidence levels, perceived impact, personal plans).

Furthermore, acceptance of the model may be biased, by attracting participants who were particularly intrigued by the unconventional method of delivery (WhatsApp®) offered by SIMBA and chose to participate. These participants might represent a population of learners who prefer the use of modern technology and are keen to participate in novel and innovative methods of learning. This sample selection bias may disproportionately tilt the positive response from respondents, leading to false belief of population acceptance of the model. Further research investigating how individual differences such as learning styles and personality play a role in acceptability and preference for the model would be interesting. Nevertheless, the results demonstrate participants’ satisfaction with the session.

### Future of SIMBA

The results from this SIMBA Adrenal session provide evidence that the delivery of SBL via social media is a promising strategy, with a potential of engaging healthcare professionals worldwide. We aim to further expand the reach of SIMBA with more frequent sessions to encourage globalisation and bridge existing gaps in healthcare. Additionally, SIMBA may be used in future credentialing and assessment processes. Thus, our vision is to achieve low-cost teaching delivery, with minimal resources required to organise each session.

## Conclusion

The SIMBA model mirrors the Kolb’s experiential learning theory from practical point of view. The results demonstrate the positive reception of SIMBA highlighting the advantages of utilising social media as a platform for medical education. Improvements in confidence levels and core competencies using existing freeware social media software paves way for such sessions to be conducted at a low cost internationally.

## Supplementary Information

**Additional file 1.**

## Data Availability

The datasets used and/or analysed during the current study are available as a supplementary file.

## Abbreviations

SBL: Simulation-based learning
LBL: Lecture-based learning
SIMBA: Simulation via Instant Messaging – Birmingham Advance
ACC: Adrenocortical carcinoma
CAH: Congenital adrenal hyperplasia
MDT: Multidisciplinary team
MCQ: Multiple-choice questions
BMNAH: Bilateral macronodular adrenal hyperplasia
ACGME: Accreditation Council for Graduate Medical Education
